# Phosphorylation of gH2AX as a novel prognostic biomarker for laryngoesophageal dysfunction-free survival

**DOI:** 10.18632/oncotarget.9172

**Published:** 2016-05-04

**Authors:** María José de Miguel-Luken, Manuel Chaves-Conde, Begoña Quintana, Alicia Menoyo, Isabel Tirado, Verónica de Miguel-Luken, Jerónimo Pachón, David Chinchón, Vladimir Suarez, Amancio Carnero

**Affiliations:** ^1^ Department of Medical Oncology, Virgen del Rocío University Hospital, Seville, Spain; ^2^ Department of Radiation Oncology, Virgen del Rocío University Hospital, Seville, Spain; ^3^ Department of Otorhinolaryngology, Virgen del Rocío University Hospital, Seville, Spain; ^4^ University of Málaga, Málaga, Spain; ^5^ Department of Pathology, Virgen del Rocío University Hospital, Seville, Spain; ^6^ Instituto de Biomedicina de Sevilla, IBIS/Hospital Universitario Virgen del Rocío/ Universidad de Sevilla/ Consejo Superior de Investigaciones Científicas, Seville, Spain

**Keywords:** laryngeal cancer, H2AX, biomarker, laryngeal preservation, DDR, Pathology Section

## Abstract

Current larynx preservation treatments have achieved an improvement of laryngoesophageal dysfunction-free survival (LDS) but lead to significant toxicities and recurrences. At present, there is no evidence to select the group of patients that may benefit from preservation approaches instead of surgery. Therefore, laryngeal biomarkers could facilitate pretreatment identification of patients who could respond to chemoradiation-based therapy. In this study, we evaluated retrospectively 53 patients with larynx cancer to determine whether gH2AX phosphorylation (pH2AX) alone or in combination with the membrane protein MAP17 (PDZK1IP1) could be used as prognostic biomarkers. We also evaluated whether the completion of cisplatin treatment and radiotherapy could predict survival in combination with pH2AX.

We found that the dose of cisplatin received but not the length of the radiotherapy influenced LDS. High-pH2AX expression was associated with prolonged LDS (HR 0.26, *p* = 0.02) while MAP17 correlated with overall survival (OS) (HR 0.98, *p* = 0.05). High-MAP17 and high-pH2AX combined analysis showed improved LDS (with 61.35 months *vs* 32.2 months, *p* = 0.05) and OS (with 66.6 months *vs* 39.8 months, *p* = 0.01). Furthermore, the subgroup of high-pH2AX and optimal dose of cisplatin was also associated with OS (72 months *vs* 38.6 months, *p* = 0.03) and LDS (66.9 months *vs* 27 months, *p* = 0.017). These findings suggest that pH2AX alone or better in combination with MAP17 may become a novel and valuable prognostic biomarker for patients with laryngeal carcinoma treated with preservation approaches.

## INTRODUCTION

Squamous cell carcinoma of the head and neck represents 4% of all cancers diagnosed worldwide, with more than 500.000 new cases recorded in 2008 [1, 2]. Of them, 30% were laryngeal cancer with an estimated age-standardized world mortality rate of 2.3/100.000 habitants. Alcohol and tobacco abuse are common etiologic factors [3] but exposure to hard-alloys dust, chlorinated solvents [4] and familiar genetic patterns [5] have been also implicated. The role of Human Papilomavirus (HPV) is well established for squamous cell carcinoma of the oropharynx but it remains unclear for laryngeal cancer [6]. Furthermore, these patients are at risk of developing second primary tumors due to chronic aerodigestive tract carcinogen exposure: 14% in 5 years, 26% in 10 years and 37% in 15 years [7]. The main prognostic factor for overall survival (OS) is tumor staging, where node invasion is more relevant than tumor extension [8]. Other OS prognostic factors are patient's comorbidity, performance status-ECOG (PS) [9], persistent toxic consumption habits [10], second primary tumor appearance [11] and primary tumor localization. In particular, glottic tumors have an 81% OS rate while supraglottic tumors drop to 70%, probably due to early detection [12]. Disease-free survival prognostic factors include PS [9, 13], node invasion [13, 14], localization [9], pathologic stage (pT) [14], surgical resection margins [13] and pretreatment tracheotomy [15]. Moreover, T4 primary extension and more than 2 cm tumoral invasion of the base of the tongue were shown to be associated with increased salvage laryngectomy in the Veterans study [16].

Total laryngectomy was the gold standard treatment by the 1980s with subsequent loss of speech and airway patency [17]. Consequently, treatment aims changed in order to improve patient's quality of life through larynx sparing approaches. Currently, early stages (I and II) are treated with either surgery or radiotherapy (RT) as they have been accepted to have similar effectiveness. However, both treatments have not been compared in a randomized trial so far. Reported five-year OS is typically 70 to 90% [18, 19]. Advanced disease requires multimodal approach, usually a combination of chemotherapy (CT) or biotherapy (B) plus RT. Although functional organ sparing approaches permit larynx preservation, they do not provide a survival advantage over total laryngectomy [20]. Three sparing approaches are accepted: RT, bio or chemotherapy with concomitant radiotherapy (B/CT RT) and induction CT (ICT) followed by RT with or without B/CT.

CTRT with concurrent cisplatin showed higher preservation rates compared to other two arms with RT alone or induction cisplatin plus fluorouracil followed by RT (88% *versus* -*vs*- 70% and 75%, respectively) with similar two and five year survival [21]. Later, a 10-year follow-up publication confirmed that the arms that included ICT improved laryngectomy-free survival (LFS). Contrary to preservation rates, LFS includes not just the need of salvage laryngectomy but also speech and swallowing quality. It is, therefore, more similar to what we currently understand as larynx preservation [22]. A subsequent meta-analysis for locally advanced larynx cancer found that adding CT concomitant with RT led to a benefit of 6.5% absolute improvement in 5-year OS [23].

The optimal dose of cisplatin during RT remains still unclear [24-26]. Two or three courses of three-weekly cisplatin could be considered the optimal dose for concurrent CTRT and equivalent doses of carboplatin have also been accepted by expert panels. However, preservation approaches entail up to a 43% rate of late toxicities [27] and have not shown to prolong OS more than surgery. Interestingly, 5-year OS was reported to drop from 67.4% in 1985 to 61.9% in 2007 (Source: Surveillance, Epidemiology and End Results Program. Accessed: http://seer.cancer.gov/). Nevertheless, these results do not allow making major conclusions, as preservation approaches were not broadly used until the time of database collection.

The high rate of toxicities and the non-improved survival with preservation approaches lead to the need for biomarker development. Predictive larynx biomarkers would facilitate pretreatment identification of those patients who are unlikely going to be cured by radiation-based therapy. By managing these patients with surgery rather than a preservation approach, local disease control and possibly survival could potentially be optimized and unnecessary treatment related morbidities from unsuccessful larynx treatments could be avoided. However, there are still no clinical or molecular biomarkers validated in standard practice at present.

γH2AX is a component of the histone octamer in nucleosomes. It is phosphorylated (pH2AX) by kinases such as ataxia telangiectasia mutated (ATM) and ATM-Rad3-related (ATR) upon DNA damage. PH2AX is involved in recruiting DNA repair proteins in response to the presence of DNA double-strand breaks (DSB) and therefore it has been studied as a biomarker of DNA damage for new drug development. As such, the presence and magnitude of pH2AX is an indication of persistent, unrepaired DNA damage [28]. pH2AX induction appears within minutes in cells after DNA damage and reaches maximum levels after 30 minutes. The repair process includes the phosphorylation of hundreds to thousands γH2AX surrounding the DSB site in order to form a focus that open the chromatin structure and serve as a platform for the accumulation of factors involved in the DNA damage response [29]. γH2AX phosphorylation has been studied as prognostic biomarker in early operable non-small cell lung cancer (NSCLC) and endometrial carcinomas. In the NSCLC study, low levels of phosphorylated γH2AX correlated with better survival outcomes. The combination of wild type p53 and low-phosphorylated γH2AX phenotype showed also better survival. In the endometrial trial, p-γH2AX positively correlated with p53 levels although the relation with survival could not be proved. However, in both studies patients were treated with surgery and not with radiotherapy [30, 31].

Our goal was to determine whether pH2AX by itself or in combination with other molecular and clinical findings could be a prognosis biomarker for laryngeal carcinomas treated with RT alone or CTRT.

## RESULTS

### Clinical cohort description

Patients were mainly male (93.7%) with squamous carcinoma histopathology (100%) and good general condition (PS 0-1 = 96.8%). Tumors were more frequently localized in the supraglottic (58.7%) and 74.6% were stage III. Nodal involvement was observed in 25.4% of the patients and 6.3% had primary T4 extension. Pretreatment tracheotomy was required for 31.7% patients. Organ preservation approaches include B or CTRT (74.6%), RT (14.3%), or ICT-B/CTRT (9.5%) (Table 1). Most of the patients received concurrent CT or B during RT (82.5%). The preferred treatment was cisplatin 100 mg/m2 on days 1, 22, and 43 of radiotherapy (73%) followed by weekly cisplatin 40 mg/ m2 (11.5%) and cetuximab (11.5%). Carboplatin was selected for only 4% of patients.

**Table 1:** Population characteristics and treatment

Population characteristics	No. %	
**Mean age**	63.7 years	
**Male**	59	93.7
**Squamous cell carcinoma**	63	100.0
**Smokers of ≥ 10 pack-years**	59	93.7
**Regular alcohol intake**	45	71.4
**PS 0-1**	61	96.8
**Pretreatment tracheotomy**	20	31.7
**Cigarette smoking**		
Current smokers	43	68.3
Former smokers	18	28.6
Never smokers	2	3.2
**Localization**		
Supraglottic	37	58.7
Glottic	24	38.1
Subglottic	2	3.2
**TNM Staging**		
II	6	9.5
III	47	74.6
IV	10	15.9
**Treatment approach**		
Surgery	1	1.6
Radiotherapy	9	14.3
B/CTRT	47	74.6
ICT-B/CTRT	6	9.5

**Figure 1:**
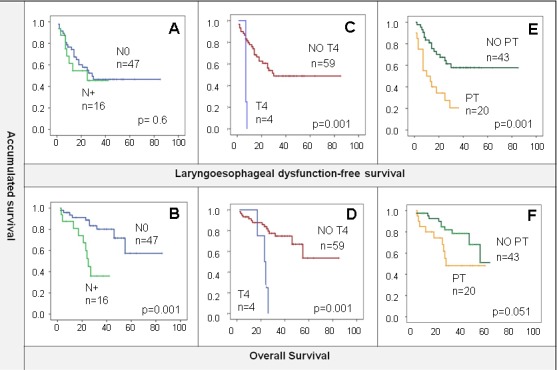
**A.** and **B.** N0 compared with N positive LDS/OS. OS was better in the patients who had no lymph node involvement. **C. and D.** T4 local tumor extension shows worse OS and LDS than the rest of patients. **E. and F.** worse LDS and OS is observed in patients who required pretreatment tracheotomy (PT).

At the time of the analysis, 20 (32%) deaths and 29 (46%) recurrences had occurred with a median follow-up of 29 months (m). Locoregional relapse occurred in 19 (30%) patients, 7 (11%) presented locoregional plus distant metastases, and 3 (4.8%) only distant metastases; of them, 14 (48.3%) were candidates for salvage surgery. Laryngoesophageal dysfunction (LD) occurred in 51% of the total; main reasons for LD were tumoral local recurrence (75%) followed by the need of a tracheostomy of feeding tube (15.6%). Mean OS was 58 m (47.7-68 m, CI 95%), LDS 46 m (36-55.5m, CI 95%), and LRC 54.6 m (44-65 m, CI 95%). Moreover, 2-year LRC rate was 63%. The 2-year cumulative proportion of patients with larynx preservation and OS were 57% and 80% respectively. Lymph node involvement was associated with worse OS (N0: 64.2m *vs* N1/2: 26.8 m, *p* < 0.01) but not with LDS (46.8 *vs* 24.6, *p* = 0.6) (Figure 1B and 1A). Tumor local extension impacted negatively on both OS and LDS (OS non-T4 60.7 m *vs* T4 22.5 m; LDS non-T4 48.5 m *vs* T4 7.3m, both *p* = .001) (Figure 1D and 1C). Furthermore, patients who required pretreatment tracheotomy (PT) had worse OS (37.2 m *vs* 61.8 m, *p* = 0.051) and LDS (19.6 m *vs* 55.4m, *p* = 0.001) (Figure 1F and 1E). Therefore, our cohort behaves similarly to others reported in the literature [16, 21].

### Cisplatin and radiotherapy as prognostic markers in larynx cancer

As per the RTOG 0129 phase III clinical trial results, patient were classified by the dose of cisplatin received during the radiation treatment. In total, 52.4% reached the optimal dose of cisplatin during radiotherapy whereas 17.5% could not reach the cisplatin optimal dose, 12.7% did not receive any radiosensitizer, and 14.3% were treated with other radiosensitizers. 3.1% were unknown.

**Figure 2:**
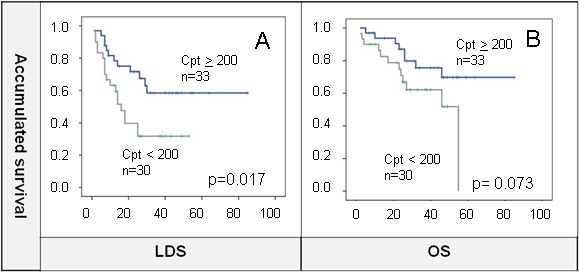
**A.** and **B.** Cisplatin (Cpt) optimal dose (≥200 mg/m2) showed significant LDS benefit that was not maintained for OS.

Cisplatin optimal dose (≥200 mg/m2) was associated with better outcomes for survival although this was statistically significant for LDS (OS: 67 m *vs* 39 m, *p* = 0.073; LDS: 56m *vs* 24 m, *p* = 0.017; LRC: 60.4 m *vs* 29 m, *p* = 0.12) (Figure 2B and 2A). Receiving an optimal dose of cisplatin showed better LDS (*p* = 0.023) than lesser doses (HR = 0.24), other radiosensitizers (HR = 0.32), and no concurrent radiosensitizers (HR = 0.65). However, this benefit was not observed for OS probably because the analysis did not take into account patients that needed salvage laryngectomy and did not preserve the organ.

**Figure 3:**
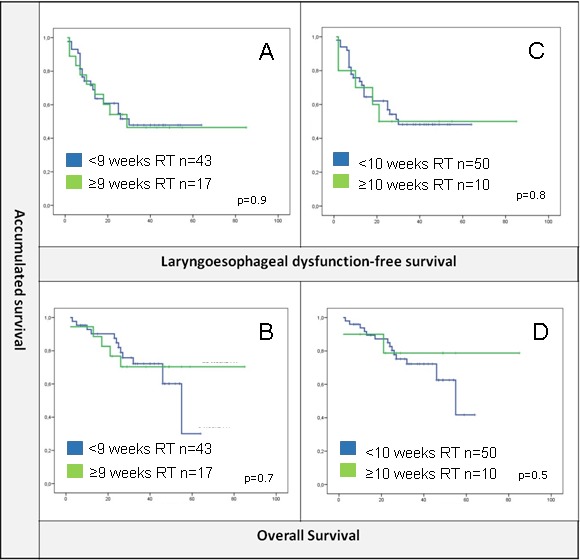
**A.** and **B.** No differences were found for radiotherapy delivered within less than 9 weeks or ≥9 weeks in terms of LDS or OS. C and D. Same results for a cut-off of 10 weeks.

On the other hand, total dose of radiation delivered was 70 Gy as per standard local guidelines. Patients that completed radiotherapy within 8 or 9 weeks were compared to those that suffered interruptions or delays but no differences were found in terms of OS or LDS between groups (Figure 3).

### pH2AX in larynx tumor samples

**Figure 4:**
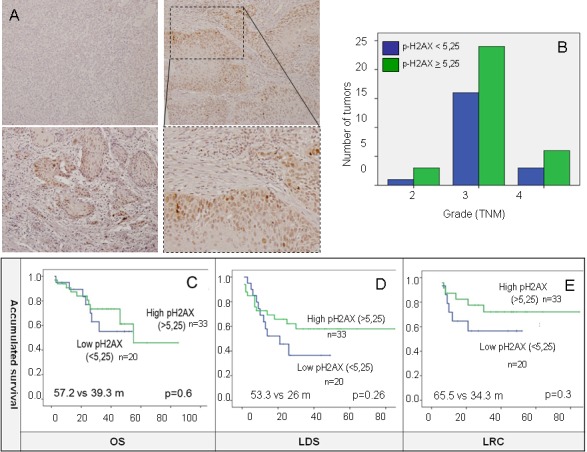
**A.** Positive pH2AX expression, considered as nuclei staining was shown in 46 (86.8%) samples. **B.** Levels of pH2AX are equally distributed among tumor stages. **C.**, **D.** and **E.** high-pH2AX show a trend towards better OS, LDS and LRC not statistically significant in the Kaplan-Meier analysis. 5.25 as indicated by the ROC curve were used as cut-off for defining high and low expression of pH2AX for survival analysis.

Out of 63 samples only 53 were analyzed for pH2AX expression either due to technical problems or because they did not contain any tumor cellularity. Positive pH2AX expression, considered as any percentage of tumoral nuclei with positive staining, was shown in 46 (86.8%) samples with a range of 1 to 70 and median expression of 10 (Figure 4A). In order to distinguish a cutoff point for pH2AX levels a ROC curve was performed and punctuation of 5.25 score chosen (supplementary Figure 1). Levels of pH2AX were equally distributed among tumor grades (Figure 4B) suggesting independence from this clinical feature, as the Chi-square test showed no differences between groups (*p* = 0.8).

When measured as a continuous variable, pH2AX had a significant positive influence with better LDS outcomes (HR 0.95, *p* = 0.02), although this was not significant for OS. As a dichotomous variable, a trend towards better OS, LDS and LRC was observed but just LDS was statistically significant in the multivariate analysis (HR 0.26, *p* = 0.02) (Table 2) (Figure 3C, 3D and 3E).

**Table 2:** LDS and OS multivariate analysis

LDS				OS			
	HR	*p*-value	CI		HR	*p*-value	CI
**PT**	1.41	0.59	0.39-5.08	**PT**	0.96	0.98	0.17-5.58
**Non-primary T4 extension**	0.09	0.00	0.02-0.49	**N negative**	0.65	0.03	0.01-0-74
**Cisplatin >200mg/m^2^**	0.09	0.00	0.02-0.43	**Cisplatin >200mg/m^2^**	0.33	0.29	0.04-2.56
**High-pH2AX**	0.26	0.02	0.09-0.78	**High-pH2AX**	0.57	0.46	0.13-2.55
**KI 67**	1.03	0.15	0.99-1.06	**KI 67**	1.06	0.038	1.00-1.12
**MAP17**	1.01	0.63	0.99-1.02	**MAP17**	0.98	0.05	0.95-1.00
**P53**	0.99	0.37	0.56-1.02	**P53**	1.03	0.12	0.99-1.07
**pERK**	0.99	0.35	0.97-1.01	**pERK**	1.01	0.65	0.98-1.04
**pAKTP**	0.99	0.41	0.98-1.01	**pAKT**	0.98	0.16	0.96-1.01

LDS: laryngoesophageal dysfunction-free survival. OS: overall survival. HR: hazard ratio. CI: confidence interval. PT: pretreatment tracheotomy. N: pathological lymph nodes.

### pH2AX and clinical findings

We also studied the potential correlation between pH2AX and clinical findings such as tumor localization, tumoral stage, smoking habit, alcohol consumption and acute toxicity development with no statistically significant association. These results suggest pH2AX to be an independent prognostic factor, as it remains significant after controlling for these variables.

### pH2AX relationship with cisplatin and radiotherapy

**Figure 5:**
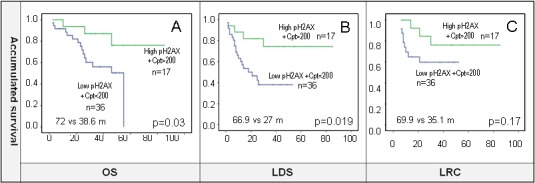
**A., B.** and **C.** pH2AX and dose of concomitant cisplatin were combined in a new variable where high-pH2AX and cisplatin (Cpt) ≥200 mg/m2 was considered as good prognosis phenotype category. The results show improved OS and LDS in this subgroup, and a trend towards better LRC. 5.25 as indicated by the ROC curve were used as cut-off for defining high and low expression of pH2AX for survival analysis.

The total dose of cisplatin was not associated with pH2AX levels (*p* = 0.4). We created a variable with two categories from pH2AX and cisplatin, in which one had a potential favorable prognosis (high-pH2AX levels, and optimal dose of cisplatin, ≥200 mg/m2), and the other unfavorable prognosis (low-pH2AX levels, and/or suboptimal dose of cisplatin < 200mg/m2 or other radiosensitizers due to the low number of patients). The favorable prognosis group correlated with increased OS, LDS (OS: 72 m *vs* 38.6 m, *p* = 0.03; LDS 66.9 m *vs* 27 m, *p* = 0.019). LRC was not statistically significant (*p* = 0.17) although there was a trend towards better outcomes in the good prognostic subgroup (69.9 m *vs* 35.1 m) (Figure 5A, 5B and 5C). Moreover, the unfavorable prognosis group correlated with worse OS (HR = 3.66, *p* = 0.044), and LDS (HR = 3.38, *p* = 0.028). LRC has a not statistically significant HR (HR = 2.4, *p* = 0.188).

We also tried to stablish whether high-pH2AX and no radiotherapy delays could impact on survival but no differences were found for both OS and LDS.

### Correlation of pH2AX with p53 and KI67

We also analyzed other markers for proliferation such as KI67 or the activated form of ERK (phosphorylated ERK, ERK-p), and apoptosis such as mutant (m) p53 or activated AKT (phosphorylated AKT, AKT-p). Our cohort showed a percentage of positive samples for ERK-p or AKT-p, but these groups did not show correlation with pH2AX expression (data not shown). KI67 in combination with pH2AX was not significant in any combination (data not shown), being pH2AX also independent of the proliferative capability of the tumor. Our results showed no correlation between p53 and pH2AX although there was a relation towards increased pH2AX with negative P53 (< 5% positive nuclei) that was not statistically significant (*p* = 0.33).

**Figure 6:**
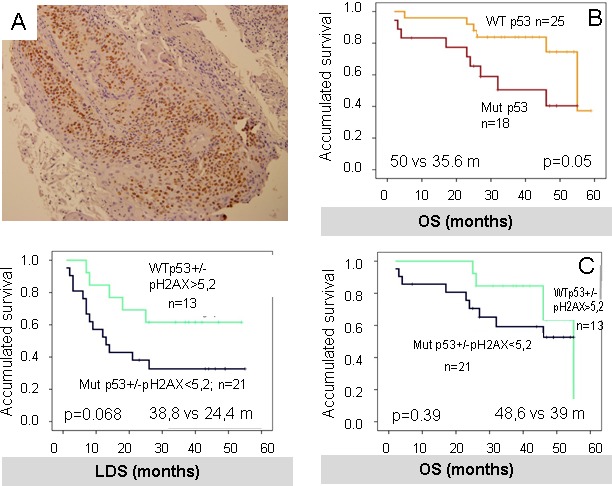
**A.** P53 was measured as >5% positive nuclei, as shown in the picture. **B.** positive P53 (+ P53) correlates with worse OS in our cohort. **C.** and **D.** results of the combination of P53 and pH2AX in a new variable. Although there was a trend towards better outcomes in the good prognosis phenotype which included negative P53 (−P53) and high-pH2AX, this was not statistically significant for OS and LDS.

However, in our cohort p53 samples positive (measured as >5% positive nuclei) (Figure 6A, + p53) correlated with worse OS (− p53 = 50 *vs* + p53 = 35.6 m, *p* = 0.047) (Figure 6B) consistent with previous literature [34].

P53 and pH2AX were combined into a new variable with the following categories: potential good prognosis phenotype (negative p53 and high-pH2AX) and unfavorable prognosis phenotype (positive p53 and low-pH2AX). Although there was an apparent relation towards better outcomes in the good prognosis phenotype, this was not significant for both OS and LDS (OS: 48.6 m *vs* 39 m, *p* = 0.39; LDS: 38.8 m *vs* 24.4 m, *p* = 0.068) (Figure 6C and 6D).

### Correlation of pH2AX and MAP17

We have recently shown that MAP17, a small non-glycosylated membrane protein overexpressed in carcinomas, expression analyzed by immunohistochemistry is associated with OS (*p* < 0.001) and LDS (*p* = 0.002) [32]. MAP17 increases endogenous ROS [35, 36]. Since ROS is a well-known mediator of DNA damage [37], we measured whether pH2AX correlated with MAP17 expression and whether the combination of both markers could strength the predictability of responses.

**Figure 7:**
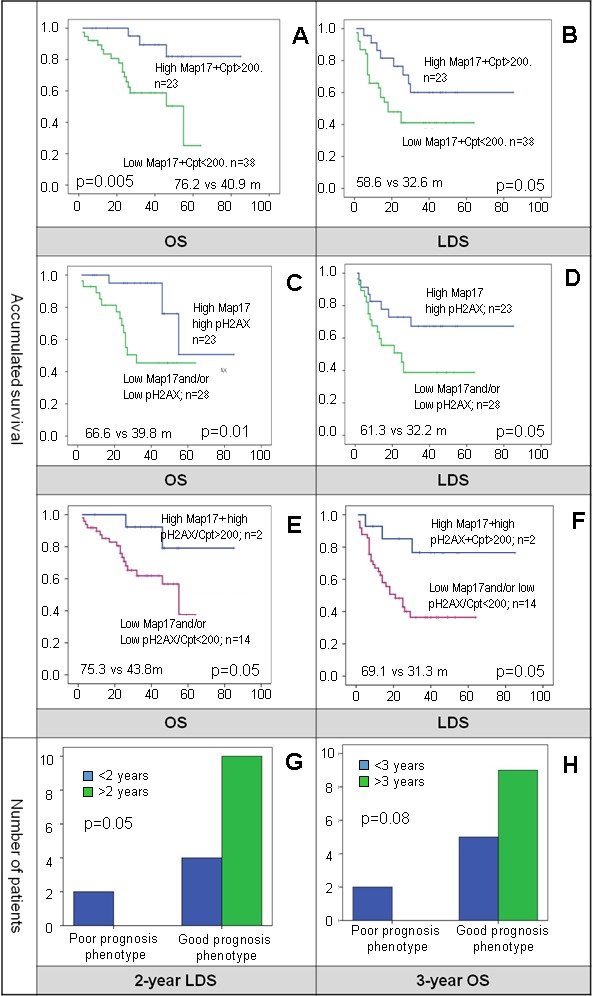
**A.** and **B.** the combination of high-MAP17 and optimal doses of cisplatin (Cpt) showed better OS and LDS. **C.** and **D.** patients with high-MAP17 and high-pH2AX with higher structural DNA-damage showed to have better OS and LDS. **E.** and **F.** survival for patients with high-pH2AX, high-MAP17 and optimal dose of cisplatin was statistically better. **G.** and **H.** the subgroup of patients with high-pH2AX, high-MAP17 and cisplatin optimal dose patients was compared to the patients that had low-pH2AX, low-MAP17 and did not complete cisplatin. Although limited in numbers, none of the patients with poor prognosis phenotype reached more than 2-years LDS or more than 3-years OS.

We found that patients with high levels of MAP17 and subject to optimal doses of cisplatin had better LDS (58.6 m *vs* 32.6 m, *p* = 0.053) and OS (76.2 m *vs* 40.9 m, *p* = 0.005) than patients with low MAP17 or not subject to optimal doses of cisplatin (Figure 7A and 7B). Furthermore, patients with high levels of MAP17 and high-pH2AX, denoting higher structural DNA-damage, conform the group of better prognosis after therapy (Figure 7C and 7D).

Moreover, patients with high-MAP17, high-pH2AX and optimal dose of cisplatin had better OS and LDS than the rest of the population (Figure 7E and 7F), and when compared with poor prognosis phenotype (low-MAP17, low-pH2AX and suboptimal cisplatin dose) (Figure 7G and 7H).

## DISCUSSION

In this manuscript, we have shown that pH2AX has a prognostic role in patients with laryngeal cancer. We hypothesize, taking into account the combined analysis with p53 and MAP17, that the DDR pathway could have an essential role in laryngeal cancer. Although further research is needed, we think that our results are opening a new window to identify biomarkers that in the future may allow changes in clinical practice, as to date there are no biomarkers that could identify those patients that will benefit from radiotherapy-based treatments instead of surgery.

We found that pH2AX was related to LDS (High-pH2AX HR 0.26, *p* = 0.02) in a cohort of 53 patients with larynx cancer. When analyzed together pH2AX expression and dose of cisplatin received during radical treatment, there is a significant correlation with survival (high-pH2AX and optimal dose of cisplatin 72 months *vs* 38.6 months, *p* = 0.03) and LDS (high-pH2AX and optimal dose of cisplatin 66.9 months *vs* 27 months, *p* = 0.019). Our data suggest that inherent DDR pathway activation (measured by the end-point of phosphorylation of H2AX) is a valuable prognostic marker in patients with laryngeal carcinoma who received preservation approaches. Our data also show the importance of performing optimal cisplatin treatment for tumor response. However, the fact that unexpected radiotherapy delays and interruptions did not affect survival in our cohort could be explained to dose compensations. Radiobiological-based calculations were performed in those patients in order to achieve an equivalent biological effectiveness by adding some more fractions to the overall treatment.

Tumor cells from clinical specimens show constitutive activation of DNA damage signalling as demonstrated by the presence of gH2AX phosphorylation and other DDR signalling proteins [38-40]. This DDR activation was found to peak at early stage tumors, persisting further among malignant tumors mostly by inactivating p53 gatekeeper [40]. It has been proposed that the DDR-network may serve as an inducible barrier to control the initial steps of tumor development by inducing p53-dependent senescence or apoptosis [38-40]. Further ongoing chronic DDR activation favours the outgrowth of malignant clones with genetic or epigenetic defects in DNA-repair mechanism such as those involved in the DDR pathway [40]. Our samples, from already malignant tumors (stages II-IV), in which only a subset of them showed mutant p53, correlated with worse onset of the disease. It is likely DNA-damage defects inducing DDR activation have been carried through the malignant process and it is possible that other proteins are mutated in the process avoiding the requirement for p53 inactivation.

pH2AH is a broad DNA damage marker that appears under different physiological conditions. Senescent cells display molecular characteristics of DNA damage [41-43]. These markers include nuclear foci of phosphorylated histone H2AX, the localization at double-strand break sites of DNA-repair and DNA-damage checkpoint factors, such as 53BP1, MDC1 and NBS1 [44-46]. Senescent cells also contain activated forms of the DNA-damage checkpoint kinases Chk1 and Chk2. During replicative senescence, markers of a DNA damage response localize at telomeres [45, 47], indicating that the DNA damage response is triggered by telomere shortening [48]. Similarly, the redox potential also results in DNA damage and senescence [49]. Very interestingly, oncogene-induced senescence has been found to induce DNA-damage due to an excess of replication forks. This oncogenic-induced hyper-replication signal, or *replication stress*, is associated with persistent DNA-damage [50, 51] inducing senescence [52]. Therefore, not only senescence is viewed as a response to DNA-damage, but DNA-damage as a marker of senescence. In that sense, high pH2AX appeared in early stage tumors and is a marker of good prognosis [38, 53, 54]. However, in our cohort, pH2AX levels are increased in advanced stages of tumors, and contrarily to this hypothesis, are a marker of bad prognosis, indicating that our pH2AX observations are not due to cellular senescence, neither by continuous proliferation nor by replication stress.

In that line, phosphorylation of H2AX is not always a marker of DNA damage. It also can be a marker of activated mTOR, eliciting replicative stress and a pseudo DNA-damage in senescent cells [46, 51, 55-58]. The dynamics of senescence exhibit 2 different steps: cell cycle arrest and further acquisition of senescence features, which includes permanent arrest, termed *geroconversion* [51, 58-60]. If geroconversion is not activated, cells are only transiently arrested with the possibility of resuming growth once the proliferation constraints have been eliminated [44, 61]. It has also been shown that if mTOR is activated under conditions of proliferative arrest, then arrest becomes permanent and the cell undergoes senescence [59, 60, 62]. Under these conditions of cell cycle arrest and mTOR activation, the phosphorylation of H2AX is launched, becoming a marker of cellular senescence [46, 52, 55, 62]. In fact, rapamycin treatment, which inhibits mTOR, can divert senescence into quiescence, allowing the cell to resume growth once conditions are more favorable [63-66]. Since mTOR is the master regulator of protein synthesis [67], it has been proposed that this contribution is due to the function of mTOR as a sensor of cellular nutrients and energy status as well as growth factor signals [68, 69]. However, it has also been reported that mTOR activation in the context of growth arrest is perceived by the cells as and unwanted oncogenic signal, activating the replicative stress and pseudo DNA-damage signaling [46, 52, 55, 62]. In any case, high levels of pH2AX as marker of cellular senescence should be associated to better prognosis, and to some extent to early stage tumors. However, it will be of interest to correlate the levels of pH2AH with those of mTOR activation in laryngeal tumors to provide a more accurate hypothesis of the pH2AH inducers.

Our data show that high levels of pH2AX correlate with better prognosis after treatment with DNA-damage agents such as cisplatin and radiotherapy, especially if cisplatin is given at optimal doses. These data are suggestive of a collaboration of DDR pathway activation, perhaps as an indicator of low DNA-repair ability and DNA-damaging agents in tumor therapy. The fact that doses of cisplatin are important for survival (Figure 2) seems to confirm this hypothesis. In line with this, wt-P53 with high levels of pH2AX conforms a subgroup of good prognosis (Figure 6) suggesting that P53 activity is essential to drive physiological response to apoptosis (or senescence) of DNA-damage agents in tumors with DDR activated.

These data are opposite to the found in early operable non-small cell lung cancer (NSCLC). In this study, low levels of pH2AX correlated with better survival outcomes. The combination of wild type p53 and low-phosphorylated γH2AX phenotype showed also better survival. However, NSCLC patients were treated with surgery and not with radiotherapy [30]. This lack of treatment with radiotherapy could be the cause of the different behavior respect the pH2AX. Radiotherapy increases oxidative stress and reactive oxygen species that in combination with preexisting DNA damage can increase cell damage above threshold inducing increased tumor efficacy. Our data support this hypothesis since combination with another ROS-inducing agent such as cisplatin is essential to gain better survival in these patients. Furthermore, the combination of MAP17, a known ROS-inducing oncogene [32, 35, 36, 70] also supports the essential role of radiotherapy in this response.

We have recently shown that MAP17 levels, a small non-glycosylated membrane protein overexpressed in carcinomas, are associated with overall survival (*p* < 0.001) and laryngoesophageal dysfunction-free survival (*p* = 0.002) [32]. MAP17 increases endogenous ROS [35, 36, 70]. ROS are well known mediators of DNA damage [37]. Our data suggest that high levels of MAP17 induced ROS that in turn increases DNA-damage and DDR signaling. Upon further DNA-damage and further increase in ROS molecules induced by cisplatin and RT treatment, tumors with higher oxidative stress (higher MAP17, higher ROS denoted by higher pH2AX), are more suitable to undergo apoptosis in the presence of P53 activity. Therefore, our data seems to confirm that pH2AX is a marker of structural DNA-damage in the laryngeal tumors that may become a novel and valuable prognostic biomarker for laryngeal carcinoma.

## MATERIALS AND METHODS

### Patient's characteristics and treatment

We evaluated 63 patients with larynx cancer from August 2005 to February 2014. However, out of the 63 tumoral samples, only 53 of them could be studied. All samples were obtained from diagnostic biopsies before any treatment. All patients completed the informed consent form and the project was approved by the local ethical committee at the HUVR (PI13/059). Patients received treatment in our institution but tumor samples were obtained from four different national hospitals where the diagnosis was made. Eligibility criteria for preservation include patients with stage II-IV laryngeal tumors that had no contraindication for chemotherapy and/or radiotherapy, significant cartilage destruction, or more than 2 cm tumoral invasion of the base of the tongue. TNM Staging System for the Larynx (7th ed., 2010) was used for tumor classification. This cohort has been previously reported in [32].

### Tissue acquirement and preparation

Formalin-fixed, paraffin-embedded tissue sections from 63 laryngeal carcinomas were selected with the collaboration of the Andalusian Health Care Biological Resource Centre. Histological characterization of all samples was done by Hematoxylin and Eosin staining, followed by immunohistochemistry (IHQ) analysis of tissue microarrays (TMA).

### Immunohistochemistry

Three-micrometer slices were sectioned from the TMA block and applied to coated, immunochemistry slides (DAKO, Glostrup, Denmark). The slides were baked overnight in a 56°C oven, deparaffinized in xylene for 20 min, rehydrated through a graded ethanol series and washed with PBS. A heat-induced epitope retrieval step was performed by heating a slide in a solution of sodium citrate buffer pH 6.5 for 2 min in a conventional pressure cooker. After heating, the slides were incubated with proteinase K for 10 min and rinsed in cool running water for 5 min. Endogenous peroxide activity was quenched with 1.5% hydrogen peroxide (DAKO) in methanol for 10 minutes, and incubation with the primary antibodies anti-gamma H2A.X (phospho S139) antibody (ab11174 from Abcam) and anti-p53: p53 FL 393 (sc-6243 from Santa Cruz); was performed for 40 min. After incubation, immunodetection was performed with the EnVision (DAKO, Glostrup, Denmark) visualization system using diaminobenzidinechromogen as the substrate, according to the manufacturer's instructions. Immunostaining was performed in a TechMate 500 automatic immunostaining device (DAKO) and measured through a double-blind visual assessment using microscopic observation according to the anatomopathological experience of pathologists. Sample scoring was performed by microscopic analysis, considering the percentage of nuclei stained cells.

### Statistical analysis and definitions

Kaplan-Meier method was used for survival analysis, using Cox Proportional Hazards model to adjust for the explanatory variables, obtain the p-values and estimate the hazard ratios (HR). Multivariate logistic regression was used to obtain odds ratio (OR) and confidence intervals (CI 95%). Pearson's correlation measured dependence between quantitative variables. A receiver operating characteristic (ROC) curve was performed to assess pH2AX cutoff point (two-year OS), which we checked using the optimal Youden index-based point. In addition, a log-rank test compared the survival distributions between high and low PH2AX both as a single variable and in combination with cisplatin optimal dose, MAP17 and p53. Categorical data were studied with contingency tables that included Chi-square statistics. Calculations were performed using SPSS 15.0 software.

OS has been defined as the length of time from diagnosis until the last medical record. LCR was measured as length of time from diagnosis until the relapse or last medical record, in those patients who did not develop distant metastases or died due to different causes than the tumor. For laryngoesophageal dysfunction-free survival (LDS) we adopted Lefebvre Larynx Preservation Consensus Panel that included as endpoint events: death, local relapse, total or partial laryngectomy, tracheotomy at two or more years, or the presence of a feeding tube at two or more years [33].

## SUPPLEMENTARY MATERIALS FIGURE


