# Supplementary material for: Phosphorylation of gH2AX as a novel prognostic biomarker for laryngoesophageal dysfunction-free survival
Source: Oncotarget. 2016 May 4;7(22):31723–37. doi: 10.18632/oncotarget.9172 (PMC5077972; doi:10.18632/oncotarget.9172)
Supplement: Supplementary file 1 [file oncotarget-07-31723-s001.pdf]

Phosphorylation of gH2AX as a novel prognostic biomarker for laryngoesophageal dysfunction-free survival

Supplementary Material

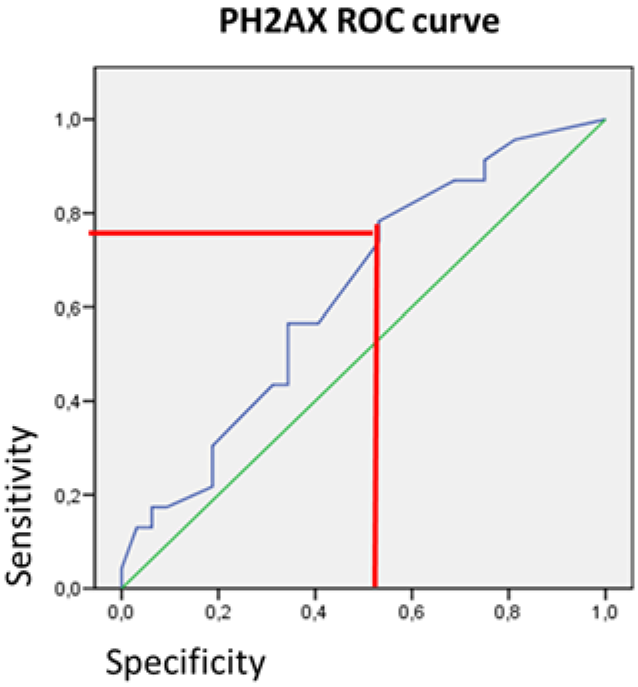

	Sensitivity	1 - Specificity
-1,0000	1,000	1,000
,5000	,957	,813
1,5000	,913	,750
2,2500	,870	,750
3,7500	,870	,688
**5,2500**	**,783**	**,531**
7,7500	,739	,531
10,5000	,565	,406
11,7500	,565	,375
13,7500	,565	,344
15,5000	,435	,344
18,0000	,435	,313
21,2500	,304	,188
23,7500	,261	,188
27,5000	,217	,188
32,5000	,174	,094
37,5000	,174	,063
45,0000	,130	,063
55,0000	,130	,031
65,0000	,043	,000
71,0000	,000	,000

Supplementary Figure 1. ROC curve to determine the pH2AX cut-off that selects which is the best point connecting sensitivity and specificity. In the graph, it is shown and the dot of the curve closer to the left superior corner.
